# Supplementary material for: Underweight Is Associated with a Higher Risk of Acute Pancreatitis in Type 2 Diabetes: A Nationwide Cohort Study
Source: J Clin Med. 2022 Sep 25;11(19):5641. doi: 10.3390/jcm11195641 (PMC9572046; doi:10.3390/jcm11195641)
Supplement: Supplementary file 1 [file jcm-11-05641-s001.zip › jcm-1877918-supplementary.pdf]

Supplementary Table S1. Adjusted hazard ratios of acute pancreatitis by BMI group according to the presence or absence of type 2 diabetes.

Diabetic Status	BMI (kg/m^2)	Subjects	Acute pancreatitis	Non-adjusted HR	Age- and sex-adjusted HR	*Adjusted HR (95% CI)
Total	< 18.5	145362	348	1.158 (1.036–1.293)	1.407 (1.259–1.571)	1.412 (1.264–1.577)
	< 23	1527723	3223	1 (Reference)	1 (Reference)	1 (Reference)
	< 25	962488	2049	1.005 (0.951–1.063)	0.861 (0.815–0.91)	0.839 (0.793–0.888)
	< 30	1137786	2897	1.202 (1.143–1.264)	1.009 (0.959–1.061)	0.927 (0.881–0.975)
	≥ 30	139137	416	1.416 (1.278–1.568)	1.377 (1.244–1.526)	1.129 (1.017–1.253)
Non-diabetes	< 18.5	140516	304	1.151 (1.023–1.296)	1.394 (1.238–1.569)	1.38 (1.226–1.554)
	< 23	1442781	2760	1 (Reference)	1 (Reference)	1 (Reference)
	< 25	875337	1688	1.004 (0.945–1.067)	0.864 (0.813–0.918)	0.857 (0.806–0.911)
	< 30	997282	2273	1.187 (1.123–1.255)	1.001 (0.947–1.059)	0.956 (0.903–1.012)
	≥ 30	114760	309	1.406 (1.25–1.582)	1.382 (1.229–1.555)	1.22 (1.082–1.375)
Diabetes	< 18.5	4846	44	1.886 (1.384–2.569)	1.874 (1.376–2.553)	1.849 (1.356–2.521)
	< 23	84942	463	1 (Reference)	1 (Reference)	1 (Reference)
	< 25	87151	361	0.741 (0.645–0.85)	0.735 (0.64–0.843)	0.725 (0.63–0.834)
	< 30	140504	624	0.785 (0.696–0.886)	0.801 (0.71–0.904)	0.77 (0.681–0.871)
	≥ 30	24377	107	0.773 (0.626–0.954)	0.882 (0.714–1.091)	0.805 (0.65–0.997)

*Adjusted hazard ratio for age, sex, alcohol consumption, smoking, type 2 diabetes, insulin administration, hypertension, dyslipidemia, chronic kidney disease, chronic pancreatitis, regular physical activity, and low income

Abbreviations: BMI, body mass index; HR, hazard ratio; CI, confidence interval

Supplementary Table S2. Incidence rates and hazard ratios of acute pancreatitis according to glycemic status and BMI category

Glycemic status	BMI (kg/m^2)	Subjects	Acute Pancreatitis	Duration (person-years)	IR	Hazard ratios (95% confidence interval)		
						Model 1	Model 2	Model 3
Normal FPG	< 18.5	121122	233	984865	0.24	1.155 (1.008–1.323)	1.396 (1.219–1.6)	1.385 (1.209-1.587)
	< 23	1160837	1963	9568228	0.21	1 (reference)	1 (reference)	1 (reference)
	< 25	644010	1098	5322932	0.21	1.005 (0.933–1.082)	0.865 (0.803–0.932)	0.859 (0.798-0.925)
	< 30	680830	1450	5626063	0.26	1.256 (1.173–1.344)	1.059 (0.989–1.133)	1.015 (0.948-1.087)
	≥ 30	73055	177	603193	0.29	1.431 (1.227–1.668)	1.389 (1.191–1.62)	1.24 (1.061-1.449)
Impaired fasting glucose	< 18.5	19394	71	152297	0.47	2.28 (1.799–2.889)	1.94 (1.531–2.459)	1.782 (1.406-2.259)
	< 23	281944	797	2297950	0.35	1.692 (1.558–1.837)	1.352 (1.244–1.468)	1.279 (1.177-1.39)
	< 25	231327	590	1899166	0.31	1.514 (1.381–1.66)	1.127 (1.027–1.236)	1.066 (0.972-1.169)
	< 30	316452	823	2601091	0.32	1.542 (1.422–1.673)	1.158 (1.067–1.257)	1.062 (0.977-1.154)
	≥ 30	41705	132	342509	0.39	1.88 (1.577–2.243)	1.649 (1.382–1.966)	1.413 (1.183-1.688)
New onset type 2 diabetes	< 18.5	2125	13	15160	0.86	4.219 (2.445–7.278)	2.737 (1.586–4.725)	2.225 (1.289-3.841)
	< 23	29619	153	234388	0.65	3.191 (2.707–3.761)	2.185 (1.852–2.578)	1.889 (1.601-2.229)
	< 25	28612	81	231155	0.35	1.711 (1.37–2.137)	1.152 (0.922–1.44)	1.013 (0.81-1.267)
	< 30	47853	172	388821	0.44	2.16 (1.848–2.524)	1.516 (1.296–1.772)	1.31 (1.119-1.534)
	≥ 30	8597	37	70119	0.53	2.578 (1.862–3.569)	2.171 (1.568–3.005)	1.759 (1.269-2.438)
Type 2 diabetes < 5 years	< 18.5	1251	15	8423	1.78	8.791 (5.294–14.599)	5.14 (3.091–8.547)	4.122 (2.478-6.857)
	< 23	24950	155	194327	0.80	3.892 (3.305–4.584)	2.347 (1.99–2.767)	2.046 (1.734-2.414)
	< 25	28756	129	230400	0.56	2.727 (2.282–3.259)	1.653 (1.382–1.978)	1.453 (1.213-1.74)
	< 30	50986	234	413100	0.57	2.757 (2.407–3.157)	1.741 (1.519–1.996)	1.499 (1.304-1.723)
	≥ 30	9692	45	78879	0.57	2.777 (2.066–3.732)	2.072 (1.542–2.785)	1.691 (1.256-2.277)
Type 2 diabetes ≥ 5 years	< 18.5	1470	16	9793	1.63	8.074 (4.937–13.204)	4.487 (2.741–7.344)	3.863 (2.359-6.326)
	< 23	30373	155	231707	0.67	3.273 (2.78–3.855)	1.856 (1.574–2.19)	1.67 (1.414-1.972)
	< 25	29783	151	233187	0.65	3.163 (2.68–3.732)	1.78 (1.506–2.104)	1.556 (1.314-1.843)
	< 30	41665	218	330621	0.66	3.218 (2.797–3.701)	1.882 (1.633–2.168)	1.654 (1.431-1.912)

≥ 30	6088	25	48339	0.52	2.525 (1.702–3.746)	1.703 (1.147–2.527)	1.425 (0.959-2.117)

Abbreviations: BMI, body mass index; IR, incidence rate (per 1000 person-years); FPG, fasting plasma glucose

Supplementary Table S3. Adjusted hazard ratios of acute pancreatitis in subjects with constant BMI

Diabetic Status	BMI (kg/m^2)	Subjects	Acute pancreatitis	Adjusted HR (95% CI)
Total	< 18.5	41917	72	1.643 (1.289-2.094)
	< 23	529269	748	1 (Reference)
	< 25	251381	368	0.84 (0.741-0.952)
	< 30	362505	696	1.037 (0.933-1.153)
	≥ 30	39820	91	1.317 (1.056-1.643)
Non-diabetes	< 18.5	41217	68	1.687 (1.313-2.167)
	< 23	513302	675	1 (Reference)
	< 25	237932	334	0.875 (0.767-0.999)
	< 30	334921	614	1.089 (0.974-1.218)
	≥ 30	35107	71	1.328 (1.037-1.701)
Diabetes	< 18.5	700	4	1.332 (0.486-3.651)
	< 23	15967	73	1 (Reference)
	< 25	13449	34	0.55 (0.366-0.827)
	< 30	27584	82	0.646 (0.469-0.89)
	≥ 30	4713	20	0.937 (0.562-1.562)

Adjusted hazard ratio for age, sex, alcohol consumption, smoking, type 2 diabetes, insulin administration, hypertension, dyslipidemia, chronic kidney disease, chronic pancreatitis, regular physical activity, and low income

Abbreviations: BMI, body mass index; HR, hazard ratio; CI, confidence interval
